# Phylogenetic history shapes the composition of floral scents in a specialized pollination mutualism

**DOI:** 10.1111/nph.71133

**Published:** 2026-03-29

**Authors:** Li Cao, Nina Joffard, Simon T. Segar, Daniel Souto‐Vilarós, Steven D. Johnson, Bruno Buatois, Anne‐Geneviève Bagnères, Martine Hossaert‐McKey, Magali Proffit

**Affiliations:** ^1^ Centre d'Écologie Fonctionnelle et Évolutive, CNRS UMR5175 Université de Montpellier, EPHE, IRD Montpellier Cedex 5 34293 France; ^2^ Yunnan Key Laboratory of Forest Ecosystem Stability and Global Change/State Key Laboratory of Plant Diversity and Specialty Crops Xishuangbanna Tropical Botanical Garden, Chinese Academy of Sciences Mengla 666303 China; ^3^ Univ.Lille, CNRS, UMR 8198 ‐ Evo‐Eco‐Paleo Lille 59000 France; ^4^ Agriculture and Environment Department Harper Adams University Newport TF10 8NB UK; ^5^ Science Research Initiative, College of Science The University of Utah Salt Lake City UT 84112 USA; ^6^ Centre for Functional Biodiversity University of KwaZulu‐Natal School of Life Sciences Pietermaritzburg 3209 South Africa

**Keywords:** chemical mediation, *Ficus*, floral scents, mutualism, phylogeny, pollination

## Abstract

- Most studies of the chemical ecology of plant–pollinator interactions emphasize the role of pollinator‐mediated selection in shaping floral scent composition. Nevertheless, phylogeny may constrain the metabolic pathways underlying these profiles, thereby influencing the evolutionary trajectory of the emitted signals.
- To investigate whether phylogenetic history constrains plant chemical communication, we used the obligate fig–fig wasp mutualism. We collected floral scents from receptive figs of 32 *Ficus* species, representing diverse lineages across tropical and subtropical regions, using dynamic headspace extraction. Chemical compositions were analyzed via gas chromatography‐mass spectrometry and evaluated for the phylogenetic signal using multivariate analyses.
- Our results revealed a strong phylogenetic signal in the volatile organic compounds (VOCs) emitted by receptive figs. Conversely, using the same analysis, we found no relationship between the scent profile and the pollinator phylogeny.
- Our findings demonstrate, across diverse *Ficus* lineages, that phylogenetic constraints play a significant role in the diversification of VOC signals emitted by receptive flowers, suggesting constraints in the biosynthetic pathways of volatile compounds.

## Introduction

An overwhelming majority (*c*. 90%) of angiosperm species are pollinated by animals (Ollerton *et al*., 2011). The success of biotic pollination depends on the production of signals by plants and their recognition by animals to facilitate partner encounter (Chittka & Thomson, 2001; Majetic *et al*., 2009; Schiestl & Johnson, 2013). One of the most predominant modalities of signaling is the emission of volatile organic compounds (VOCs) by plants and their olfactory detection by animals (Raguso, 2008; Hossaert‐McKey *et al*., 2010; Byers, 2021). Floral scents are usually mixtures of numerous VOCs, produced by several biosynthetic pathways in different compartments of the cell, such as cytosol or plastids (Pichersky *et al*., 2006; Muhlemann *et al*., 2014; Picazo‐Aragonés *et al*., 2020), resulting in more or less complex floral phenotypes (Raguso *et al*., 2015). A remarkable diversity of VOCs has been documented; more than 1700 floral VOCs had already been identified in plants more than a decade ago (Knudsen *et al.*, 2006) and this number is steadily increasing (Knudsen & Gershenzon, 2020; Dötterl & Gershenzon, 2023). VOCs belong to four basic biochemical classes: terpenoids (mainly monoterpenes or sesquiterpenes), derivatives of fatty acids, benzenoids/phenylpropanoids and other amino acid derivatives (Muhlemann *et al*., 2014; Dötterl & Gershenzon, 2023). Floral scents vary among species in their qualitative composition, in the proportions of different compounds in the blend and in their overall concentrations. These sources of variation can influence the attractiveness of scents across a range of pollinators (Raguso, 2008; Majetic *et al*., 2009; Schiestl & Johnson, 2013) as well as the specificity of the interactions (Hossaert‐McKey *et al*., 2010). However, our understanding of the evolution of floral scent chemistry is still limited due to insufficient information about the roles played by phylogenetic history vs pollinator‐mediated selection (Levin *et al*., 2003; Delle‐Vedove *et al*., 2017; Farré‐Armengol *et al*., 2020; Liu *et al*., 2024).

It is generally assumed that the composition of floral scents is shaped by strong selective pressures (Schiestl & Johnson, 2013; Friberg *et al*., 2019; Opedal *et al*., 2022), including pollinator‐mediated selection (Darwin, 1877; Majetic *et al*., 2009; Junker & Parachnowitsch, 2015; Schiestl *et al*., 2018; Moré *et al*., 2021). A key line of evidence for this idea is convergence in scent composition among unrelated species sharing the same guild of pollinators (Knudsen & Tollsten, 1995). Floral VOCs are thus a component of pollination syndromes (Dellinger, 2020). For instance, the combination of benzaldehyde, lilac aldehyde and phenylacetaldehyde is typical of moth‐pollinated flowers (Dötterl *et al*., 2006; Moré *et al*., 2021). Likewise, dimethyl disulfide is a prominent scent constituent in bat‐pollinated species in the New World (Knudsen & Tollsten, 1995; Von Helversen *et al*., 2000), although it is also found in hundreds of fly‐pollinated plants (Zito *et al*., 2015). Floral VOCs involved in pollinator attraction can also be subjected to other biotic selection pressures, for example, those exerted by herbivorous insects (Segar *et al*., 2019). Considering plant chemistry as a whole, the evolution of volatile compounds to attract mutualistic partners, such as pollinators, and their patterns of expression in plants, are not independent of the production of other compounds involved in plant defense against antagonists (Burkle & Runyon, 2016; Rusman *et al*., 2018; Kantsa *et al*., 2025). In addition to biotic selective pressures, changes in environmental conditions mainly due to global change (e.g. elevated temperature, drought and tropospheric ozone) can also affect the emission of floral scents, disrupting existing adaptations and favoring compounds whose properties are better suited to local environmental conditions (Farré‐Armengol *et al*., 2014; Campbell *et al*., 2019; Jaworski *et al*., 2022; Dubuisson *et al*., 2024) or even leading to a complete suppression of detectable scent emissions under extreme heat stress (Cordeiro & Dötterl, 2023).

The numerous examples of evolutionary convergence in floral volatiles driven by pollinator‐mediated selection (Darwin, 1877; Schiestl *et al*., 2011, 2018; Junker & Parachnowitsch, 2015; Liu *et al*., 2024) reinforced a general consensus that the composition of floral volatiles is highly homoplasious (Raguso *et al*., 2006). However, recent studies have detected some phylogenetic signal (i.e. ‘tendency for related species to resemble each other more than they resemble species drawn at random from the tree’ (Blomberg & Garland, 2002)) in the distribution of floral VOCs across different species of diverse lineages, where closely related species tend to have similar floral VOC composition (Jürgens, 2004; Steiner *et al*., 2011; Prieto‐Benítez *et al*., 2016; Joffard *et al*., 2020; Cna'ani *et al*., 2021; Liu *et al*., 2024).

Among the processes postulated to underlie phylogenetic signal in floral VOC profiles is phylogenetic constraint (Delle‐Vedove *et al*., 2017), that is any component of the phylogenetic history of a lineage that obscures a pattern based on predictions of adaptive hypotheses (McKitrick, 1993). For example, because flowers of bat‐pollinated plants often emit dimethyl disulfide (Knudsen & Tollsten, 1995), a plant that shifts from bird pollination to bat pollination would be expected to evolve the ability to synthesize this compound, thus converging with other bat‐pollinated plants. While this expectation is borne out in most cases (Domingos‐Melo *et al*., 2023), some plants that have made this switch have not evolved this ability (e.g. scentless flowers, such as those of some *Heliconia* spp.; Knudsen & Tollsten, 1995), suggesting that some aspect of their evolutionary history has prevented this response, and implying that pollinator attraction in these species must rely on other signals (e.g. visual or tactile). Few studies invoking phylogenetic constraints on floral VOC evolution have tried to examine the underlying mechanisms (Delle‐Vedove *et al*., 2017). Regarding constraints, some lineages may simply lack the specific biosynthetic pathways required to produce certain classes of VOCs (e.g. similar to the exclusive production of betalains instead of anthocyanins in Caryophyllales). Additionally, competition for major building blocks among different pathways in the biosynthesis of VOCs and other specialized metabolites make it difficult for plants to run multiple competing pathways (Majetic & Sinka, 2013). Finally, evaluating the relative importance of pollinator‐mediated selection and phylogenetic constraints is further complicated by the heterogeneous nature of floral bouquets, which often include vegetative compounds, antiherbivore defenses and microbial artifacts alongside pollinator‐attracting VOCs.

Phylogenetic niche conservatism (PNC) is another process that could result in phylogenetic signal (Losos, 2008). PNC is tendency of closely related species to retain similar ecological niches (e.g. pollination systems), which can be assessed by comparing observed trait similarity to expectations under a Brownian‐motion model of evolution. PNC is especially likely in highly derived lineages, such as *Ficus*, in which evolutionary reversals (e.g. from specialized syconia to open, *Dorstenia*‐like inflorescences) appear to be unlikely (Herre *et al*., 2008; Segar *et al*., 2025). For example, *Ficus* species from the same section tend to be pollinated by congeneric species of agaonid wasps (Agaonidae; Cruaud *et al*., 2012). While this PNC is not absolute (i.e. sister species of figs are often not pollinated by sister species of agaonids), it does produce a phylogenetic signal if we consider *Ficus*/pollinator associations at the global level (Cruaud *et al*., 2012). This suggests that pollinator olfactory responses to VOCs are likely to exhibit phylogenetic signal, whether driven by innate sensory constraints or coevolutionary tracking of host scents, reinforcing the phylogenetic patterns observed in host‐pollinator associations.

There are many open questions in investigations of the role of evolutionary history in shaping the evolution of profiles of floral VOCs. First, to what extent does the composition of VOCs within a lineage show a phylogenetic signal? To date, cases of convergent evolution in VOC profiles driven by pollinator‐mediated selection, indicating only a weak phylogenetic signal, have attracted most attention (Liu *et al*., 2024). Indeed, most studies have investigated related plants pollinated by very different guilds of pollinators, where divergent selection on floral odors is expected to be strong (Friberg *et al*., 2019; Farré‐Armengol *et al*., 2020). Pollinator effects might thus be so strong that they mask or swamp the effects of phylogeny (Steiner *et al*., 2011). As noted by Peakall *et al*. (2010), assessing phylogenetic signal in the evolution of floral scents might be easiest in systems in which closely related plants are pollinated by specific pollinators from a single guild.

Second, even in the cases where convergence has been shown (indicating a weak phylogenetic signal), it often involves a small number of VOCs. Whether other components of the plants' VOC profiles show a stronger phylogenetic signal appears to be rarely investigated (Delle‐Vedove *et al*., 2017; Joffard *et al*., 2020).

Third, what processes underlie phylogenetic signal in floral VOC composition? Are there phylogenetic constraints on the biosynthesis of floral VOCs? Also, are there phylogenetic constraints on olfactory responses of pollinators to VOCs?

Several features in the species‐specific relationship between the fig and fig/wasp mutualism make it especially well‐suited for examining questions about phylogenetic signal in the composition of floral VOCs and phylogenetic constraints on pollinator‐mediated selection. In this highly specialized coevolved system, species specificity is largely mediated by floral scents emitted by receptive figs (Proffit *et al*., 2008, 2020; Chen *et al*., 2009; Wang *et al*., 2013; Souto‐Vilarós *et al*., 2018). Generally, intraspecific stability in scent composition, particularly when a species is associated with a single pollinator, facilitates species‐level macroevolutionary analyses. Furthermore, the diversity of these interacting lineages (*c*. 800 species of both *Ficus* and their wasps; Segar *et al*., 2025) offers an adequate sample size for phylogenetic comparative analysis. Finally, we have reasonably well‐resolved phylogenies for a subset of both figs and wasps (Cruaud *et al*., 2012).

**Fig. 1:**
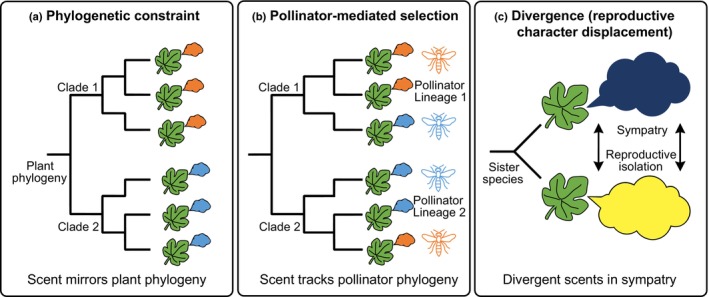
Conceptual framework of alternative evolutionary scenarios shaping floral scent composition in the *fig*‐wasp mutualism. Different colors represent distinct chemical scent profiles. (a) Phylogenetic constraint hypothesis: scent evolution is strictly conserved. Closely related *Ficus* species (e.g. within the same clade) exhibit similar scent profiles (e.g. orange vs medium blue) due to conserved biosynthetic pathways, independent of their pollinators. (b) Pollinator‐mediated selection hypothesis: scent composition is driven by pollinator preference. Scent profiles group according to specific pollinator lineages (represented by matching colored wasp icons) rather than the plant phylogeny. Unrelated plants pollinated by related wasps converge on similar scents. (c) Divergence (reproductive character displacement) hypothesis: driven by the need for reproductive isolation. Closely related species, particularly those in sympatry, evolve highly divergent scent profiles to prevent pollinator visitation errors and hybridization. The deep blue and bright yellow colors in this panel represent sharply contrasting chemical profiles, distinct from the examples in (a) and (b), emphasizing the extreme divergence required for effective isolation in sympatry.

In this study, we present a large dataset from 32 fig species (over half of which is reported here for the first time), many with overlapping geographical distributions. We use this data set to examine the effects of factors that may have shaped the composition of fig VOCs over the circa 75 Ma evolutionary history of the genus (Cruaud *et al*., 2012). We address the following questions: (1) Are the volatile‐chemical profiles of receptive figs from each species unique? (2) Is there a phylogenetic signal in the traits comprising *Ficus* floral VOC profiles? (3) If there is such a phylogenetic signal, do phylogenetic constraints contribute to it? For example, do differences in the chemical profiles of receptive figs of species belonging to different sections result from intrinsic constraints associated with VOC biosynthesis? And finally (4) is VOC profile similarity between fig species correlated with phylogenetic proximity of their respective pollinators? If so, this could indicate constraints on pollinator olfactory responses to VOCs. The conceptual framework illustrating these questions and the expected evolutionary scenarios (e.g. phylogenetic constraint vs pollinator‐mediated selection) is presented in Fig. 1.

## Materials and Methods

### Studied species

VOCs emitted by receptive figs (i.e. figs ready to be pollinated) were collected from 3 to 33 individuals per species from 32 different *Ficus* species (242 individuals in total) distributed mainly in the tropical regions of five continents (see Table 1 for sampling details and taxonomic authorities) between 2002 and 2020. These species (15 monoecious and 17 dioecious) were selected in order to represent the different clades of *Ficus* (13 of the 19 sections from the five subgenera), the phylogenetic relationships of which have already been published (Cruaud *et al*., 2012). Our approach was to acquire a sufficiently large dataset in terms of species richness and within‐species replication.

**Table 1:** Sampled species, reproductive systems, years of collection, sampling localities and number of samples collected for each species.

Taxa/Species	Pollinator species	Reproductive system	Year of collection	Sampling locality (country, region)	Sample size	References
Subgen. Pharmacosycea						
Sect. Pharmacosycea						
*F. maxima* Mill.	*Tetrapus americanus*	M	2009	France (French Guiana)	3	This study
Sect. Oreosycea						
*F. hombroniana* Corner	*Dolichoris sp*.	D	2016	Papua New Guinea, Madang	3	This study
Subgen. Urostigma						
Sect. Malvanthera						
*F. macrophylla* Desf. ex Pers.	*Pleistodontes froggatti*	M	2009	USA, Hawaii	4	This study
Sect. Americana						
*F. aurea* Nutt.	*Pegoscapus jimenezi*	M	2009	USA, southern Florida	3	This study
*F. citrifolia* Mill.	*Pegoscapus tonduzi*	M	2011	USA, southern Florida	7	This study
Sect. Galoglychia						
*F. lutea* Vahl	*Allotriozoon heterandromorphum*	M	2008	South Africa, Kwazulu‐Natal	4	This study
*F. glumosa* Delile	*Elisabethiella glumosa*	M	2007–2009	South Africa, Kwazulu‐Natal	5	Cornille *et al*. (2012)
*F. trichopoda* Baker	*Elisabethiella bergi*	M	2008	South Africa, Kwazulu‐Natal	3	This study
*F. burkei* (Miq.) Miq.	*Elisabethiella burkei*	M	2008	South Africa, Kwazulu‐Natal	5	Cornille *et al*. (2012)
*F. natalensis* Hochst.	*Alfonsiella longiscapa*	M	2008	South Africa, Kwazulu‐Natal	15	Cornille *et al*. (2012)
Subgen. *Ficus*						
Sect. *Ficus*						
*F. carica* L.	*Blastophaga psenes*	D	2020	France, Occitanie	6	This study
*F. erecta* Thunb.	*Blastophaga nipponica*	D	2010	China, Taiwan	7	This study
Sect. Eriosycea						
*F. fulva* Reinw. ex Blume	*Valisia compacta*	D	2002	Brunei, North Borneo	6	Hossaert‐McKey *et al*. (2016)
Subgen. Sycidium						
Sect. Sycidium						
*F. asperifolia* Miq.	*Kradibia gestroi afrum*	D	2007	UK, Greenhouse Univ. Leeds	4	This study
*F. cyrtophylla* (Wall. ex Miq.) Miq.	*Kradibia sp*.	D	2009	China, Yunnan	3	This study
*F. exasperata* Vahl	*Kradibia gestroi afrum*	D	2003–2005	India, Western Ghats	33	Borges *et al*. (2008)
Sect. Palaeomorphe						
*F. subulata* Blume	*Kradibia subulatae*	D	2009	China, Yunnan	4	This study
*F. tinctoria* G. Forst.	*Kradibia rutherfordi*	D	2005, 2010	China, Yunnan & Taiwan	7	This study, Proffit *et al*. (2009)
Subgen. Sycomorus						
Sect. Adenosperma						
*F. itoana* Diels	*Ceratosolen armipes*	D	2016	Papua New Guinea, Madang	11	Souto‐Vilarós *et al*. (2018)
*F. adenosperma* Miq.	*Ceratosolen cf adenospermae*	D	2016	Papua New Guinea, Madang	3	Souto‐Vilarós *et al*. (2018)
*F. microdictya* Diels	*Ceratosolen kaironkensis*	M	2016	Papua New Guinea, Madang	10	Souto‐Vilarós *et al*. (2018)
*F. mollior* F. Muell. ex Benth.	*Ceratosolen medlerianus*	D	2016	Papua New Guinea, Madang	4	This study
Sect. Sycomorus						
*F. racemosa* L.	*Ceratosolen fusciceps*	M	2005–2006	China, Yunnan	5	Soler *et al*. (2011)
*F. oligodon* Miq.	*Ceratosolen cf emarginatus*	D	2008	China, Yunnan	3	This study
*F. auriculata* Lour.	*Ceratosolen emarginatus*	D	2008	China, Yunnan	5	Hossaert‐McKey *et al*. (2016)
*F. sycomorus* L.	*Ceratosolen galili*	M	2008	South Africa, Kwazulu‐Natal	4	Proffit & Johnson (2009)
*F. botryoides* Baker	*Ceratosolen blommersi*	M	2007	Madagascar	3	This study
Sect. Hemicardia						
*F. semicordata* Buch. Ham. ex Sm.	*Ceratosolen gravelyi*	D	2006–2007	China, Yunnan	10	Chen *et al*. (2009)
Sect. Sycocarpus						
*F. fistulosa* Reinw. ex Blume	*Ceratosolen constrictus*	D	2006	Brunei, Northern Borneo	11	Hossaert‐McKey *et al*. (2016)
*F. hispida* L.f.	*Ceratosolen sp*.	D	2003	India, Western Ghats	31	Hossaert‐McKey *et al*. (2016)
*F. septica* Burm.f.	*Ceratosolen sp*.	M	2013	Philippines	10	Conchou *et al*. (2014)
*F. nota* (Blanco) Merr.	*Ceratosolen notus*	M	2013	Philippines	10	Conchou *et al*. (2014)

D, Dioecious; *F*., *Ficus*; M, Monoecious; Sect., Section; Subgen., Subgenus.

### Collection of floral VOCs

VOCs were collected using dynamic headspace sampling from either intact figs (attached to trees in the field) or detached figs (excised in the field or glasshouse). For intact sampling, care was taken to exclude leaves from the enclosures, which contained only the target syconia and a small portion of the supporting branch. To correct for vegetative background emissions and ambient noise, control samples were simultaneously collected from similar branch sections lacking both leaves and syconia. Two different collection methods were used based on two different traps, containing either 30 mg of Alltech Super Q adsorbent (ARS Inc., Gainesville, FL, USA; until 2009) or 3 mg of a 1 : 1 mixture of Tenax‐TA 60–80 and Carbotrap 20–40 (from 2010 onward). Comparative studies have shown that the type of trap does not significantly influence volatile acquisition (Hossaert‐McKey *et al*., 2016). Furthermore, previous tests on several *Ficus* species found no significant differences in the volatile blends emitted by figs on trees in the field and figs detached from trees, with VOCs collected on the field site or transported to a nearby laboratory (Proffit *et al*., 2008). Some data used in this study were previously published, as indicated in Table 1.

### Analysis of floral VOCs

Used traps were stored in the dark at −20°C until analysis. Samples were analyzed at the ‘Platform for Chemical Analyses in Ecology’ (PACE), technical facilities of the LabEx CeMEB (Centre Méditerranéen pour l'Environnement et la Biodiversité, Montpellier, France), using a gas chromatography (GC, Trace™ 1310, Thermo Scientific™, Milan, Italy) coupled to a mass spectrometer (ISQ™ QD Single Quadrupole; Thermo Scientific™, Milan, Italy). Samples were consistently analyzed using methods described elsewhere (see Cornille *et al*., 2012, Hossaert‐McKey *et al*., 2016, and Souto‐Vilarós *et al*., 2018 for details). Compound identification was based on computer matching of the mass spectra with the NIST MS library, on retention indices reported in the literature (Adams, 2007) and on retention indices obtained by injection of reference compounds, when these were available. By comparing the spectrum of each sample with that of the respective control sample (empty bag, same day and conditions of collection), putative contaminant compounds were subtracted from our chromatograms.

### Dataset on floral VOCs from receptive figs

For each compound, the percentage of the total peak area in each sample was calculated as an estimate of relative amount (semiquantitative data). This approach was chosen to focus on the evolution of signal composition (quality and ratios), which is the primary driver of species specificity and to normalize for variations in total emission intensity driven by fig size or environmental conditions during the long‐term sampling period. Only compounds that were present in at least 50% of the individuals of at least one species were considered. These represented a total of 216 VOCs (Supporting Information Table S1).

The relative proportions of all the compounds emitted by each of the 242 individuals of the 32 *Ficus* species were compiled into a dataset to facilitate the comparison of VOCs composition among different *Ficus* species.

Due to phylogenetic data existing only at the species level, mean values of the replicates (individuals within one species) available for scent composition were calculated for each species. From this, we built several distinct datasets tailored for our specific hypotheses. To test for phylogenetic signal (K_mult_ tests) and to perform phylogenetic principal component analysis (pPCA), two datasets were constructed: (1) a dataset containing major compounds only (i.e. compounds representing at least 5% of the blend in at least one species, that is 54 VOCs); (2) a dataset containing all compounds, both major and minor (i.e. all 216 VOCs). This dual approach allowed us to assess whether phylogenetic patterns are driven by the most abundant compounds alone or by the entire chemical profile.

Following the insight that secondary chemical variation can be effectively analyzed by coding for biosynthetic pathways (Barkman, 2001), and to test the hypothesis that major evolutionary lineages within *Ficus* have diverged in their overall allocation to different biosynthetic pathways, another dataset was built on the relative amounts of VOCs (major compounds only) belonging to the different chemical classes (terpenoids, fatty acid derivatives, benzenoids/phenylpropanoids and other amino acid derivatives) found in species of the 13 *Ficus* sections from five *Ficus* subgenera. Because 5 of the 13 sections were represented by only a single species, precluding a meaningful assessment of within‐group variation, they were excluded before the PERMANOVA. Thus, the final dataset used to test for among‐section variation comprised 8 sections.

### Molecular phylogenetic dataset

In order to reconstruct phylogenetic trees for the species of *Ficus* and Agaonidae included in this study, we used the published tree reconstructed using 200 *Ficus* and associated Agaonidae species (Cruaud *et al*., 2012). Although a more recent genomic phylogeny for *Ficus* is available (Gardner *et al*., 2023), we utilized the framework by Cruaud *et al*. (2012) because it (1) provides matched phylogenies for both *Ficus* and their pollinating wasps, which is essential for our comparative analyses, and (2) includes a larger proportion of the specific species sampled in our study. The relationships among the sections sampled here are congruent between the two frameworks. In Cruaud *et al*. study, *Ficus* phylogeny was reconstructed using five genes: ITS (891 bp), ETS (528 bp), glyceraldehyde 3‐phosphate dehydrogenase (G3pdh, 769 bp), chloroplast‐expressed glutamine synthetase region (ncpGS, 1630 bp) and granule‐bound starch synthase (waxy region, 1734 bp). To infer phylogenetic relationships between Agaonidae species, Cruaud *et al*. (2012) combined two nuclear protein‐coding genes (F2 copy of elongation factor‐1a (EF1a, 516 bp), Wingless (Wg, 403 bp)); two mitochondrial protein‐coding genes (cytochrome c oxidase subunit I (COI, 1536 bp), cytochrome b (Cyt b, 749 bp)) and two ribosomal genes (28S rRNA (D2‐D3 and D4‐D5 expansion regions, 1520 bp), 18S rRNA (variable regions V3‐5, 787 bp)).

### Data analysis

All data analyses were performed in R (v. 4.0.2; R Development Core Team; http://www.R‐project.org) on the two previously described datasets.

To compare volatile profiles among different *Ficus* species, we performed a permutational multivariate analysis of variance (PERMANOVA; Anderson, 2001) using the function ‘adonis’ in the package vegan (Oksanen *et al*., 2022) on the dataset of relative amounts of each VOC emitted by each individual. Data were standardized using Min–Max normalization (via the decostand function with method = ‘range’) before a PERMANOVA was conducted on the Bray–Curtis dissimilarity matrix with 999 permutations.

Then, to compare patterns in VOC pathways among the eight *Ficus* sections or in the five *Ficus* subgenera for which more than one species was sampled, we performed a PERMANOVA on the dataset compiling relative amounts of each chemical class (terpenoids, fatty acid derivatives, benzenoids/phenylpropanoids and other amino acid derivatives) for each section and subgenus. PERMANOVAs were run on the Euclidean distance index with 9999 permutations per analysis.

We further tested for the presence of a phylogenetic signal in the VOCs emitted by figs using the K_mult_ test, which is an extension of the *K* statistic of Blomberg *et al*. (2003) for multivariate traits (Adams, 2014). The multivariate Blomberg's *K* (K_mult_; Blomberg *et al*., 2003) was used to test for a significant phylogenetic signal in the multivariate VOCs dataset. This multivariate analysis allows for the detection of significant phylogenetic signal in multivariate traits whose dimensionality exceeds the number of species examined (Mitteroecker *et al*., 2025). Before the analysis, the all‐compounds dataset was centered log ratio transformed. We also used pPCA, which creates a principal component analysis (PCA)‐like ordination while controlling for phylogenetic covariance (Revell, 2009). In the pPCA analysis, the phylogenetic resemblance in a complex set of continuous variables (semiquantitative dataset) is graphically summarized by ordination of the main principal components (PCs), that is the components with the highest (high variance and strong positive autocorrelation) and the lowest (high variance and strong negative autocorrelation) eigenvalues (Jombart *et al*., 2010). The first global component (GPC1) denotes the ‘global structure’ and reveals the VOCs that are more similar in related than in distant species. The ‘local structure’ is depicted in the first local component (LPC1), which reveals the VOCs that create dissimilarities among closely related species. In the pPCAs, we used the measure of phylogenetic proximity underlying the test of Abouheif (1999) because of its utility for detecting phylogenetic signal (Pavoine *et al*., 2008). pPCA was implemented in the adephylo package (Jombart & Dray, 2009). In addition, in order to track possible effects of pollinator‐mediated selection on the pattern of VOCs emitted by receptive figs, we conducted the same analyses (i.e. K_mult_ and pPCA) on the chemical dataset using the phylogeny of the 32 associated agaonid wasp species.

## Results

### Specificity of the signal of VOCs in *Ficus*

**Fig. 2:**
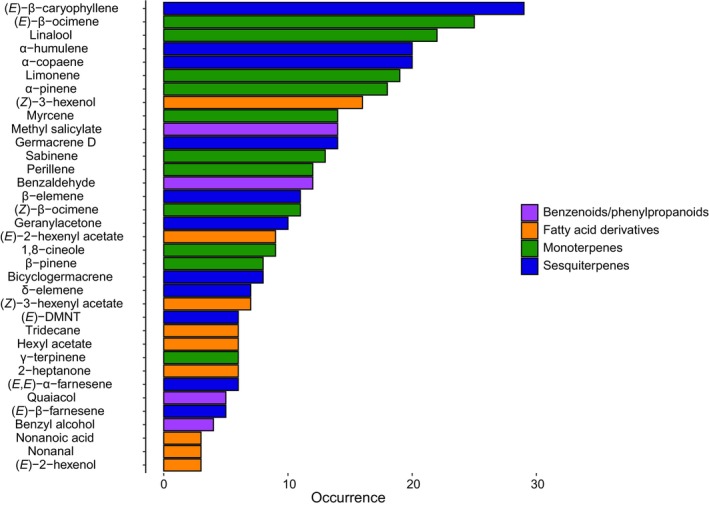
Occurrence of the major volatile compounds (i.e. compounds representing at least 5% of the blend in at least one of the sampled species) emitted by receptive figs among the 32 *Ficus* species studied. (*E*)‐DMNT, (*E*)‐4,8‐dimethyl‐1,3,7‐nonatriene.

Receptive figs of our 32 focal species produced a highly diverse collection of compounds (Table S1). In total, 216 volatile compounds were considered as representative of the VOCs profile of these species (i.e. present in at least 50% of individuals of at least one species) and retained for the statistical analysis. The mean number of compounds found per species was 19.51 ± 10.55 (mean ± SD). Receptive figs emitted floral scents comprising compounds from four chemical classes: terpenoids, fatty acid derivatives, benzenoids/phenylpropanoids and other amino acid derivatives (Fig. 2). Among these, several compounds commonly found in floral scents were detected in high proportions in the floral bouquet of almost all studied species, such as the sesquiterpenes (*E*)‐β‐caryophyllene (> 90%), α‐copaene and α‐humulene, and the monoterpenes (*E*)‐β‐ocimene (> 75%) and linalool. By contrast, only one species, *F. semicordata*, produced a compound that is uncommon among floral VOCs in this genus, 4‐methyl anisole (see Table S1 for more details).

The floral blend was generally dominated by one or a few common compounds, the identity and proportion of which differed among species. As a result, for each of the 32 fig species we analyzed, variation within species was lower than variation among species, indicating significant chemical differentiation among species. Crucially, this pattern of high intraspecific stability was consistent even in species with the highest sampling intensity (e.g. *F. exasperata*, *n* = 33, or *F. hispida n* = 31), suggesting that the core chemical profile is robustly captured even at smaller sample sizes. This species specificity is confirmed by the PERMANOVA showing that the volatile‐chemical profiles of the receptive figs are significantly different among the 32 species (*F*_31,210_ = 10.83, *R* ^2^ = 0.62, *P* = 0.001).

### Shifts in chemical class allocation among *Ficus* lineages in the VOCs emitted by receptive figs

**Fig. 3:**
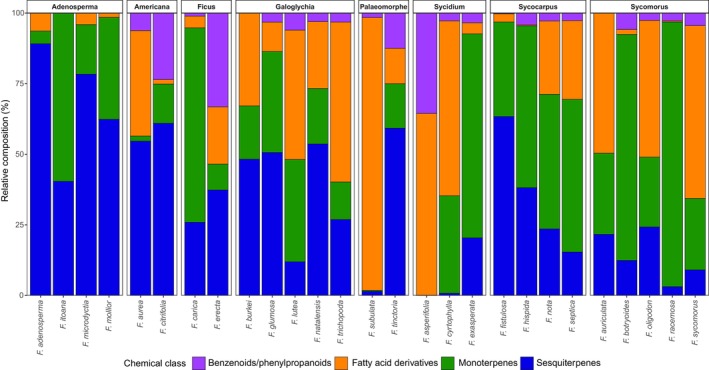
Relative composition of the four main chemical classes of floral scents across different *Ficus* sections. The stacked bar chart illustrates the relative proportions of sesquiterpenes (blue), monoterpenes (green), fatty acid derivatives (orange) and benzenoids/phenylpropanoids (purple) in the volatile profiles of the studied species. Species are grouped according to their taxonomic section (indicated by the labels at the top). Permutational multivariate analysis of variance (PERMANOVA) revealed a significant divergence in chemical class composition at the section level (*F*_7,19_ = 1.94, *R* ^2^ = 0.42, *P* = 0.042). Note that taxonomic sections represented by only a single species were excluded from the PERMANOVA analysis and are therefore not presented in the figure. *F*., *Ficus*.

We investigated whether broad evolutionary patterns in scent composition exist at higher taxonomic levels. Specifically, we tested whether different *Ficus* sections or subgenera differed in the relative importance of the four main chemical classes (e.g. terpenoids vs benzenoids). We found a significant effect of the *Ficus* section (*F*_7,19_ = 1.94, *R* ^2^ = 0.42, *P* = 0.042) on the relative amounts of the chemical classes (Fig. 3). However, no significant differentiation was detected at the subgenus level (*F*_4,27_ = 1.08, *R* ^2^ = 0.14, *P* = 0.401). This suggests that while scent chemistry is conserved within sections, these patterns do not extend to the broader subgenus level.

### Phylogenetic signal in the emission of volatile compounds by receptive figs of different species

Our analysis indicated a strong phylogenetic signal in VOC emission with significant K_mult_ values for both the major compounds (54 VOCs; K_mult_ = 0.499, *P* = 0.004) and all‐compounds (216 VOCs; K_mult_ = 0.418, *P* = 0.001) datasets. These results suggest a correlation between phylogenetic and chemical distances of floral scent in the *Ficus* species studied here.

**Fig. 4:**
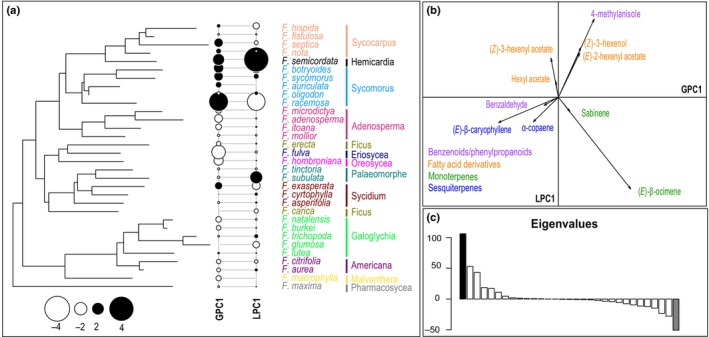
Phylogenetic principal component analysis (pPCA) of major volatile organic compounds (VOCs) emitted by receptive *Ficus* species. (a) Phylogeny of the *Ficus* species studied and results of the pPCA for major VOCs emitted by receptive figs. Positive and negative scores on GPC1 (similar VOC composition among related species) and LPC1 (dissimilar VOC composition among related species) are indicated by black and white circles, respectively. Symbol size is proportional to absolute score values. The section, as well as each species of the section, are indicated with different colors. (b) Loading of the main compounds for the global and local principal components. Only the compounds with the highest loadings (net value higher than 0.1) on the PCs are represented. Different chemical classes are indicated with different colors. (c) Barplot displaying the corresponding eigenvalues of GPC1 (black) and LPC1 (gray). *F*., *Ficus*; GPC, global principal component; LPC, local principal component; PC, principal component.

For our first analysis, focusing on major compounds only, the global distribution of the compounds on the two main axes of the pPCA, GPC1 (strong positive autocorrelation) and LPC1 (strong negative autocorrelation), is presented in Fig. 4a. These two components, GPC1 and LPC1, explain 41.06% and 28.42% of the variation in positive and negative autocorrelation, respectively. The global distribution of compounds on the pPCA was strongly correlated with the different biosynthetic pathways. For example, GPC1 (VOCs that are shared between related species) contrasts monoterpenoids (positive loading) to sesquiterpenoids (negative loading) and LPC1 (VOCs that are different between related species) opposes terpenoids (negative loadings for both mono‐ and sesquiterpenoids) to fatty acid‐derived compounds (positive loadings).

Reflecting this strong phylogenetic signal, receptive figs of species belonging to the closely related sections *Sycomorus*, *Sycocarpus* and *Hemicardia* (e.g. respectively *F. racemosa*, *F. septica* and *F. semicordata*) released high proportions of the same monoterpene, (*E*)‐β‐ocimene (positive loadings on GPC1; Fig. 4b). Likewise, species of the related sections *Eriosycea* and *Oreosycea*, as well as section *Galoglychia*, emitted a high proportion of the sesquiterpene (*E*)‐β‐caryophyllene (negative loading on GPC1; Fig. 4b).

By contrast, LPC1 underlined some differences between related species emitting contrasting amounts of particular volatile compounds. This is, for example, the case of *Ficus subulata* (section *Palaeomorphe*; positive score on LPC1; Fig. 4b), where floral volatiles were composed of high relative amounts of the fatty acid derivative, (*Z*)‐3‐hexenyl acetate. By contrast, its closest relative (same section) among the species sampled, *F. tinctoria* (negative score on LPC1; Fig. 4b), did not emit this compound. It is noteworthy that samples for both species were collected from detached figs in the field under identical conditions. The absence of this compound in *F. tinctoria*, despite similar handling, confirms that the high abundance of (*Z*)‐3‐hexenyl acetate in *F. subulata* represents a species‐specific chemical trait rather than a generic artifact of wounding. Similar differences were found between species of the section *Sycidium*: *Ficus exasperata* (negative score in LPC1; Fig. 4b) emitted high relative amounts of the monoterpene, sabinene, whereas its closest relatives among the species sampled, *F. asperifolia* and *F. cyrtophylla* (positive scores in LPC1; Fig. 4b), did not emit this terpene.

**Fig. 5:**
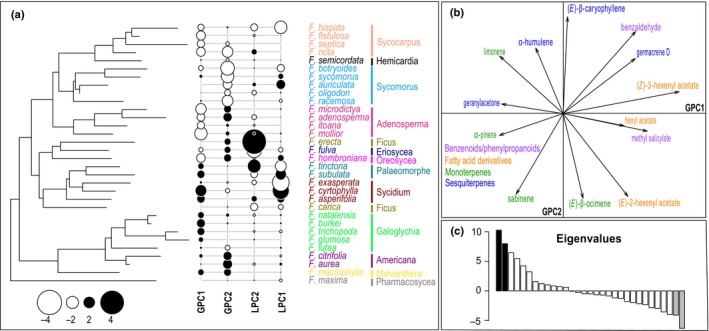
Phylogenetic principal component analysis (pPCA) of all volatile organic compounds (VOCs) emitted by receptive *Ficus* species. (a) Phylogeny of the *Ficus* species studied and results of the pPCA for all VOCs emitted by receptive figs. Positive and negative scores on GPC1–GPC2 (similar VOC composition in related species) and LPC2–LPC1 (dissimilar VOC composition among related species) are indicated by black and white circles, respectively. Symbol size is proportional to absolute score values. The section, as well as each species of the section, are indicated with different colors. (b) Loading of the main compounds for the global and local principal components. Only the compounds with the highest loadings (net value higher than 0.2) on the PCs are represented. Different chemical classes are indicated with different colors. (c) Barplot displaying the corresponding eigenvalues of GPC1–GPC2 (black) and LPC2–LPC1 (gray). *F*., *Ficus*; GPC, global principal component; LPC, local principal component; PC, principal component.

In the second analysis, which included all 216 compounds, the phylogenetic signal became significantly more diffuse, although it remained statistically significant (K_mult_ = 0.418, *P* = 0.001). This was evident in the sharp decrease in variance explained by the pPCA; the primary global and local axes (GPC1 and LPC1) explained only 23.15% and 16.68% of the variation, respectively (Fig. 5a). In order to explain as much variation in the diversity of floral VOCs as possible, we also kept GPC2 (which explained 18.10% of the variation in positive autocorrelation) and LPC2 (10.89% of the variation in negative autocorrelation; Fig. 5c). These four PCs together thus explained 41.25% and 27.57% of the variation in positive and negative autocorrelation, respectively (Fig. 5a). The inclusion of minor compounds resulted in less variation being explained in the chemical space compared with the analysis of major compounds alone. Despite this less variation being explained, the analysis revealed finer‐scale patterns not apparent before. For instance, it uniquely linked section *Sycocarpus* to the emission of other monoterpenes, such as α‐pinene, limonene and sabinene (negative loadings on GPC1; Fig. 5b), and associated sections *Malvanthera* and *Americana* with the production of benzenoid/phenylpropanoids (positive loading on GPC1; Fig. 5b).

However, within sections, some closely related species emitted contrasting relative amounts of compounds and therefore showed quite different scores on LPC2 and LPC1. For example, *Ficus tinctoria* from section *Palaeomorphe* (positive score in LPC2; Fig. 5) emitted high relative amounts of the sesquiterpene (*E*, *E*)‐α‐farnesene, whereas its closest relative among the species sampled, *F. subulata* (negative score in LPC2; Fig. 5a), did not emit this compound. *Ficus exasperata* of section *Sycidium* (negative score in LPC1; Fig. 5a) emitted high relative amounts of the monoterpene α‐pinene, whereas its closest relatives among the species sampled, *F. asperifolia* and *F. cyrtophylla* (positive scores in LPC1; Fig. 5a), did not emit this compound but emitted high relative amounts of the fatty acid derivative 2‐heptanone.

### Phylogenetic signal of the pollinator species on volatile emission by figs

**Fig. 6:**
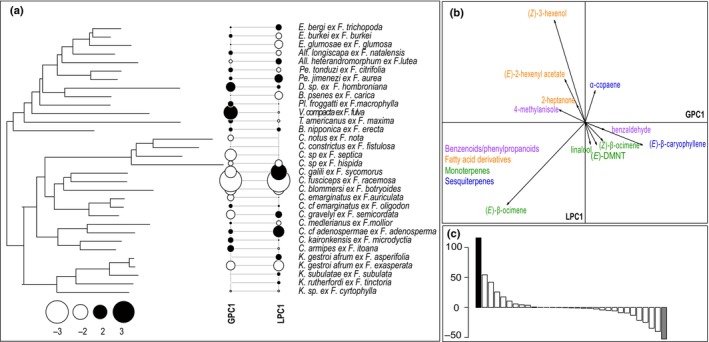
Phylogenetic principal component analysis (pPCA) of volatile organic compounds (VOCs) emitted by receptive *Ficus* species and their association with pollinator phylogeny. (a) Phylogeny of the associated pollinators of the *Ficus* species studied and results of the pPCA for major VOCs emitted by receptive figs. Positive and negative scores on GPC1 (more similar VOCs) and LPC1 (dissimilarities among related species) are indicated by black and white circles, respectively. Symbol size is proportional to absolute value. (b) Loading of the main compounds for the global and local principal components with all the compounds. Only the compounds with highest loadings (net value higher than 0.2) on the PCs are represented. The chemical classes to which VOCs belong are indicated with different colors. (c) Barplot displaying the corresponding eigenvalues of GPC1 (black) and LPC1 (gray) with all the compounds. *F*., *Ficus*; (E)‐DMNT, (E)‐4,8‐dimethyl‐1,3,7‐nonatriene; GPC, global principal component; LPC, local principal component. Pollinator genus abbreviations: *Alf*., *Alfonsiella*; *All*., *Allotriozoon*; *B*., *Blastophaga*; *C*., *Ceratosolen*; *D*., *Dolichoris*; *E*., *Elisabethiella*; *K*., *Kradibia*; *Pl*., *Pleistodontes*; *Pe*., *Pegoscapus*; *T*., *Tetrapus*; *V*., *Valisia*.

In contrast to the results for the fig species discussed above, tests for signals of the phylogeny of the 32 associated agaonid wasp species on floral scents of their hosts were not significant in either analysis (all compounds: K_mult_ = 0.349, *P* = 0.904; major compounds only: K_mult_ = 0.382, *P* = 0.588; Fig. 6). None of the PCs in pPCA were associated with a specific group of pollinating wasps.

## Discussion

In this study, we highlight the remarkable diversity of floral scent at receptivity among *Ficus* species, with 216 compounds emitted by receptive figs of the 32 focal species. Moreover, our large dataset demonstrates high within‐species similarity and strong interspecies divergence of floral scent. As shown in previous studies on smaller sample size (Grison *et al*., 1999; Proffit *et al*., 2009; Proffit & Johnson, 2009; Cornille *et al*., 2012; Hossaert‐McKey *et al*., 2016; Souto‐Vilarós *et al*., 2018), the chemical signal emitted by receptive figs is species‐specific. This specificity is likely a crucial prezygotic barrier limiting host identification mistakes by wasps. Because tropical forests and savannas often harbor many sympatric *Ficus* species, we suggest that selection drives high constancy of complex odor blends at the species level. This in turn ensures fidelity of wasps (Hossaert‐McKey *et al*., 2010; Okamoto & Su, 2021) and maintains species boundaries. Crucially, underlying this species‐level diversity, our study demonstrates for the first time on a broad scale that the evolution of these scents is also shaped by a strong phylogenetic signal, revealing a deep, shared evolutionary history.

### Specificity of the signal among fig species

Among the floral VOCs emitted, terpenoids were dominant (see Table S1 for total relative amounts of each chemical class per species). But surprisingly, in this highly specific mutualistic interaction, the floral signal comprises a mixture of compounds that are ubiquitous among angiosperms, such as (*E*)‐β‐ocimene, linalool and (*E*)‐β‐caryophyllene (in different forms, such as enantiomers, oxides), that are found in the floral VOCs of 71, 70 and 52% of angiosperm species, respectively (Knudsen *et al*., 2006). While bioassays are required to confirm whether other species rely strictly on blends of common compounds, only *F. semicordata* in our study employs a ‘private channel’ strategy. Its signal is dominated by an uncommon compound for *Ficus* (4‐methylanisole), the function of which in attracting the specific pollinating wasp was confirmed by Chen *et al*. (2009). The evolution of this qualitatively distinct signal, a distinctive exception in our dataset, could be explained by primary non mutually exclusive hypotheses including: (1) extreme selection for reproductive isolation in a community with high signal noise from congeners or (2) a contingent but successful biosynthetic innovation in this specific evolutionary branch. Distinguishing between these possibilities would require further integrative phylogenetic and ecological investigation, combined with a better understanding of the underlying biosynthetic pathways. Breaking through metabolic conservatism may represent an extreme adaptive innovation under phylogenetic constraints imposed by specific biosynthetic pathways. Nevertheless, experimental evidence from model species indicates that the blend of floral VOCs emitted may rely on precise proportions of each major compound, each in a precise enantiomer configuration, to elicit specific attraction (Chen & Song, 2008; Proffit *et al*., 2020).

The species specificity of VOC blends observed in this study aligns with the findings of other studies (Proffit *et al*., 2009; Souto‐Vilarós *et al*., 2018), supporting the hypothesis that chemical signal differentiation among sympatric *Ficus* species likely acts as the primary barrier limiting pollinator mistake visits. Indeed, when VOC similarity between closely related species belonging to the same species group exceeds a threshold, the risk of pollinator sharing increases significantly (Wang *et al*., 2013, 2016; Deng *et al*., 2022). However, when phylogenetic constraints limit the divergence of volatile profiles between closely related species, reproductive isolation may rely on multimodal filters. In these cases, ecological traits, such as microhabitat differentiation (Cornille *et al*., 2012) or physical and tactile barriers – specifically ostiole morphology and surface contact cues (Wang *et al*., 2013; Souto‐Vilarós *et al*., 2018; Segar *et al*., 2025) – function as essential ‘fail‐safe’ mechanisms to prevent hybridization when long‐range chemical attraction is insufficient. Conversely, such incomplete chemical divergence may have facilitated important host shifts recorded in the evolutionary history of *Ficus* (Segar *et al*., 2025).

### Systemic constraints are a key factor shaping floral odors of figs

True phylogenetic signal is most easily detected when a large number of taxa are available. In this study, we successfully compiled a comprehensive dataset from 32 *Ficus* species representing different subgenera, despite the significant logistical challenges of locating trees at the ephemeral receptive stage (individual fig trees in general flower one to several times a year and stay at receptive stage for a maximum of 10–15 d; Proffit *et al*., 2008). The 32 fig species and their associated pollinators included in this study offered a more than adequate sample size for phylogeny‐based analyses.

In support of our hypothesis concerning phylogenetic constraints on the diversification of VOCs in specialized pollination interactions, the multivariate analysis on semiquantitative data of flower scents clearly demonstrates that the variation in VOC composition across *Ficus* species is partly explained by phylogenetic signal. This is further supported by the significant differentiation in chemical class allocation observed at the section level, confirming that closely related lineages tend to rely on similar biosynthetic pathways. This conservatism in VOC production likely stems from genetic constraints in the biosynthetic pathways of volatile compounds. The inherent architecture of metabolic pathways can restrict the feasibility of ‘chemical innovation’, forcing species to rely on modifications of existing biosynthetic pathways (e.g. via shifts in enzyme activity or regulation leading to concentration ratio adjustments or stereoisomer conversions) to adapt to new ecological pressures. Our finding that *Ficus* sections differ significantly in their relative proportions of major chemical classes reinforces this hypothesis. Our results provide a clear example of this constraint: VOCs of the closely related section *Sycomorus* and *Sycocarpus* are primarily composed of monoterpenes (e.g. (*E*)‐β‐ocimene), whereas other related sections (*Eriosycea*, *Oreosycea*) predominantly emit sesquiterpenes (e.g. (*E*)‐β‐caryophyllene), suggesting that divergence often occurs within, rather than between, major biosynthetic pathways. However, the lack of significant differentiation at the subgenus level suggests that while biosynthetic conservatism is strong within sections, homoplasy (e.g. convergent evolution) may obscure these patterns over deeper evolutionary timescales.

This phylogenetic inertia is likely amplified by chemical pleiotropy linking vegetative and reproductive traits. For instance, *Ficus* species produce diverse pentacyclic triterpenes in their latex as a defense against herbivores, and these compounds share the isoprenoid pathway with the lower molecular mass volatile terpenoids (monoterpenes and sesquiterpenes) used to attract pollinators (Tetali, 2019). Consequently, evolutionary changes in investment toward defensive metabolites may constrain or influence the diversity of floral scents. As argued by Segar *et al*. (2019), this linkage implies that compounds are subjected to stabilizing selection due to their dual role in both mutualist attraction and antagonist defense, acting as an evolutionary brake that tethers compound evolution to ancestral metabolic frameworks. Indeed, recent genomic and chemical analyses of Malagasy figs revealed a moderate (but significant) phylogenetic correlation between syconial and leaf chemodiversity (Nguyen *et al*., 2025), supporting the hypothesis that selective pressures on vegetative defense can indirectly shape the floral volatile profiles of reproductive structures through shared biosynthetic constraints.

### Signal complexity – differentiation between major and minor compounds

The inclusion of numerous minor compounds in our analysis statistically reduced the strength of the phylogenetic signal (K_mult_ values decreased from 0.499 to 0.418) compared with the strong signal driven by the major constituents of the floral blend. However, this statistical dilution does not imply that minor compounds are biologically irrelevant. While they constitute ‘statistical noise’ that obscures deep phylogenetic trends, these minor components – or specific ratios between them – likely represent recent, species‐specific adaptations. Biological evidence supports diverse isolation strategies: *Ficus semicordata* uses a single dominant compound (4‐methylanisole) for specific attraction (Chen *et al*., 2009), whereas in *Ficus carica*, the specific blend and ratios of major and minor compounds are required to elicit pollinator attraction (Proffit *et al*., 2020). This suggests that scent evolution in *Ficus* operates on two tiers. The strong, overarching phylogenetic patterns are defined by evolutionary shifts in a few dominant, often widespread compounds, such as (*E*)‐β‐ocimene and (*E*)‐β‐caryophyllene. By contrast, the hundreds of minor compounds are often species‐specific or erratically distributed, likely representing species‐level adaptive fine‐tuning. Indeed, experimental evidence has shown that the specific pollinator attraction relies on specific blends combining major compounds with key minor constituents (Proffit *et al*., 2020). However, while broad phylogenetic signals and general attraction are carried by major biosynthetic pathways, minor compounds often prove integral to the rigorous ‘chemical filters’ that ensure precise pollinator specificity (Clavijo McCormick *et al*., 2014) and reproductive isolation, particularly when major compounds are shared across species.

### The missing signal on pollinator phylogeny – a cautious interpretation of an unexpected result

Surprisingly, and in contrast to the strong signal from the host‐plant phylogeny, we found no significant association between VOC composition and the phylogeny of the pollinating wasps. Our finding that plant phylogeny explains scent composition, whereas pollinator phylogeny fails to explain scent preference, points to a distinct evolutionary dynamic in the *Ficus*–pollinator mutualism. Specifically, this discrepancy implies that closely related pollinators do not retain similar olfactory preferences. We propose that this pattern is driven by strong selection for prezygotic isolation among sympatric species. Since many *Ficus* species and their pollinators share overlapping geographical ranges and phenologies, divergence in olfactory preference is crucial to prevent cross‐attraction and hybridization (Proffit *et al*., 2009). Consequently, pollinator preferences likely undergo rapid divergence, where sister species evolve distinct chemical search images to ensure specific host recognition. Indeed, specialized pollinators are known to evolve strong adaptive innate preferences for specific host signals (Schiestl & Johnson, 2013).

Mechanistically, this rapid behavioral divergence is likely facilitated by the high evolutionary lability of insect chemosensory systems. Genomic studies on Hymenoptera, including fig wasps, have revealed that gene families encoding odorant receptors (ORs) and odorant‐binding proteins (OBPs) evolve at accelerated rates through rapid duplication, deletion and positive selection (Robertson & Wanner, 2006; Xiao *et al*., 2013). This genomic plasticity allows pollinators to swiftly ‘tune’ their sensory apparatus to novel or minor host compounds, effectively decoupling their olfactory preferences from their phylogenetic history.

Beyond these biological explanations, caution is warranted in interpreting this negative result. The absence of a detectable signal in our analysis does not rule out the role of specific attractants, but indicates a lack of phylogenetic congruence, suggesting that closely related pollinators do not necessarily track similar floral scents. A potential methodological reason for this finding is that our analysis included the full range of VOCs identified in the floral headspace, rather than focusing only on those compounds that are key attractants for fig wasps. The signal from a few critical compounds could have been masked by the noise from many nonbioactive ones (Mant *et al*., 2005).

Demonstrating the mechanism of pollinator‐driven selection conclusively would require a multi‐faceted approach. Future studies could employ electrophysiology recordings to identify the key bioactive compounds (Dötterl *et al*., 2006), followed by behavioral assays (e.g. Y‐tube olfactometry) to confirm wasp preferences for specific host blends as performed for *Ficus carica* (Proffit *et al*., 2020). With this approach, phylogenetic signal was detected in the antennal response profiles of euglossine bee species to the attractive volatile compounds emitted by perfume‐rewarding plants (Brandt *et al*., 2021). Ultimately, a gene‐for‐gene approach, linking the evolution of scent‐producing genes in *Ficus* with olfactory genes in their pollinators, would provide the definitive evidence for coevolutionary selection on this chemical communication channel.

### Placing the *Ficus* system in a broader evolutionary context

Using the unique fig/fig wasp system, we have gained valuable insights into the evolutionary trajectory of floral scents in a nursery pollination mutualism, finding a strong phylogenetic signal. Phylogenetic signal has also been detected in diverse pollination systems, although its interplay with selection varies. For instance, in Nyctaginaceae, the systematic utility of floral fragrances was demonstrated, showing that scent composition reflected phylogenetic relationships (Levin *et al*., 2003). Similarly, in *Silene* (Caryophyllaceae), the composition of floral VOCs emitted during the night was found to be associated with a stronger phylogenetic signal than that of VOCs emitted during the day, likely owing to a simpler guild of nocturnal pollinators and less pollinator‐mediated selection for divergence (Prieto‐Benítez *et al*., 2016). In the sexually deceptive orchids of the genus *Ophrys* (section *Pseudophrys*), floral scents appear to be shaped both by phylogenetic constraints and by pollinator‐mediated selection, depending on the chemical class (Joffard *et al*., 2020). By contrast, only a weak, nonsignificant trend toward phylogenetic signal was observed for the perfume flowers of orchids in two subtribes (*Catasetinae* and *Stanhopeinae*), owing to the rapid evolution of divergent chemistries between these closely related subtribes pollinated by male euglossine bees (Liu *et al*., 2024). In the Australian deceptive orchid genus *Chiloglottis*, pollinated by specific wasps, no correlation between chemical and phylogenetic distances was identified (Peakall *et al*., 2010).

Furthermore, the strong chemical conservatism we observed aligns with studies on geographic variation within *Ficus*. While intraspecific variations in VOCs have been documented in fig trees associated with different pollinator species across their ranges (Rodriguez *et al*., 2017; Deng *et al*., 2023), there is evidence for stability in scent composition in widespread *Ficus* species that are pollinated by a single species throughout their distribution (Soler *et al*., 2011). It is possible that in some cases for this mutualism, stabilizing selection to maintain communication with a specific pollinator partner (Moe *et al*., 2012) often overrides geographically localized selective pressures imposed by heterogeneous environmental conditions or local variations in antagonist communities. This results in the conservation of ancestral traits (PNC), thereby reinforcing the phylogenetic signal in scent profiles.

However, we acknowledge that ecological factors likely interact with these evolutionary constraints. As demonstrated in *Brassica rapa*, biotic selective pressures (e.g. herbivory) can significantly alter floral chemical profiles, mediating tradeoffs between pollinator attraction and defense mechanisms (Schiestl *et al*., 2014). In parallel, within the *Ficus* system, sympatric species may undergo chemical divergence to facilitate pollinator isolation – a process driven by reproductive character displacement and to some extent herbivore avoidance (Volf *et al*., 2018). In the present dataset, heterogeneity in sampling intensity prevented a robust statistical test of geographical or habitat‐driven patterns. However, the specific impacts of these ecological factors remain to be fully disentangled. We suggest that future studies, employing targeted sampling designs across conspecific populations in sympatry and allopatry, should specifically investigate how ecological forces might modulate the phylogenetic baselines established here.

### Conclusions

In conclusion, our results on fig floral VOCs provide further insights into the processes by which floral VOCs diversify. Our findings suggest that diversification frequently involves transitions between branches of the same biosynthetic pathway (as described by Raguso, 2014), rather than leaps across pathways, consistent with a stepwise evolutionary pattern. Although our sampling of 32 species across the genus provided a robust foundation for phylogenetic analysis, future work expanding this taxonomic coverage will continue to refine these patterns, despite the logistical challenge of locating trees at the ephemeral receptive stage.

Beyond broad‐scale sampling, addressing current gaps in our understanding of cross‐pathway gene functional changes in nonmodel plants is necessary for a precise mechanistic elucidation of these evolutionary transitions. Therefore, future research should prioritize resolving the functional divergence among members of VOC‐related gene clusters (e.g. terpene synthases; Zhou & Pichersky, 2020) to map these genetic mechanisms onto the observed chemical phenotypes. This will further unravel the ‘stepwise evolution’ of VOCs. Furthermore, to fully disentangle phylogenetic constraints from environmental noise, intensified studies under semi‐controlled conditions, such as common garden experiments, are crucial. Quantifying the limits of phenotypic plasticity in this manner is essential to assess the adaptive potential of these specialized interactions, particularly in the face of rapid environmental shifts associated with global climate change (Memmott *et al*., 2007; Burkle *et al*., 2013).

## Competing interests

None declared.

## Author contributions

MH‐M and MP designed the research. MH‐M, LC, MP, STS, DS‐V and SDJ performed field volatile collections. BB, MP, DS‐V and LC were responsible for the GC‐MS analysis. MP, NJ, LC and STS analyzed the data. MH‐M, LC and MP wrote the paper. A‐GB, NJ, SDJ and STS edited the paper. MH‐M and MP are co‐last authors.

## Disclaimer

The New Phytologist Foundation remains neutral with regard to jurisdictional claims in maps and in any institutional affiliations.

## Supporting information


**Table S1** A diverse collection of compounds produced by the receptive figs of our 32 focal species.
Please note: Wiley is not responsible for the content or functionality of any Supporting Information supplied by the authors. Any queries (other than missing material) should be directed to the *New Phytologist* Central Office.

## Data Availability

The data that support the findings of this study are available in the Supporting Information of this article (Table S1). The phylogenetic relationships used in the comparative analyses were derived from previously published data (Cruaud *et al*., 2012).
